# Leaving space for imagination: mental imagery-informed teaching framework for enhancing imagery vividness in creative writing

**DOI:** 10.3389/fpsyg.2026.1767208

**Published:** 2026-04-20

**Authors:** Weiwei Yao, Mohd Nizam Saad, Osama Khalid Radwan Shatnawi, Yuying Yang

**Affiliations:** 1 College of Arts and Science, Universiti Utara Malaysia, Sintok, Malaysia; 2 College of Fine Arts, Langfang Normal University, Langfang, China

**Keywords:** cognitive load, creative writing, educational psychology, higher education, imagery vividness, instructional design, mental imagery, pedagogical intervention

## Abstract

Currently, many multimodal materials, such as pictures, videos, and audio, are used in undergraduate creative writing classes. However, students still commonly feel a “lack of visual imagery” during the ideation process and difficulty in arousing emotions. Psychological research indicates that mental imagery is a crucial internal simulation mechanism supporting creative output. Nevertheless, higher education rarely sets “vividness of mental imagery” as an explicit teaching objective. Based on this, this study proposes a mental imagery-oriented teaching framework, “leaving space for imagination,” which systematically guides students to generate and refine multisensory mental imagery by controlling the cue modality and the level of explanatory explicitness. A single-group pre-test and post-test design was adopted with 100 undergraduates majoring in media- and film-related fields at a Chinese university. The core of the class was the “four-stage lesson structure”: introduction and goal-focusing, single-modality cueing, mental imagery generation and initial writing, and work sharing and imagery-focused refinement. The Vividness of Visual Imagery Questionnaire and Plymouth Sensory Imagery Questionnaire were measured before and after the teaching unit. Pre–post changes were analysed using paired-samples *t*-tests, with paired-design effect sizes estimated using Cohen’s *dz*. Paired-samples *t*-tests indicated pre–post increases in VVIQ, *t*(99) = 18.45, *p* < 0.001, ΔM = 11.48, 95% CI (10.25, 12.72), and PSIQ total, *t*(99) = 14.93, *p* < 0.001, ΔM = 20.34, 95% CI (17.64, 23.04), with large paired-design effect sizes (Cohen’s *d*z). All seven PSIQ sensory subscales (visual, auditory, olfactory, gustatory, tactile, bodily sensation, and emotional imagery) also increased from pre-test to post-test. Overall, the findings provide preliminary evidence that a low-specification, single-modality cueing approach can be integrated into regular undergraduate creative writing instruction and is associated with increased self-reported imagery vividness. Future work should incorporate comparison groups, product-quality assessment, and process measures to test transferability and longer-term effects on creative outputs.

## 1 Introduction

In recent years, capabilities such as “creativity,” “higher-order thinking,” and “cross-media narrative” have been frequently emphasized in international and national educational policies (Barton et al., 2023, p. 1318; Batty et al., 2019, p. 68; Hague, 2024; Vincent-Lancrin et al., 2019). In response, creative writing has been more systematically incorporated into university curricula and talent-development plans, particularly as a foundational module for students’ future content production and narrative practice (Zhang, 2023). Classroom practice usually relies on case analysis, model dissection, audio-visual clips, and task-driven writing training, and is often described as “experiential” and “multimodal” (Barton et al., 2023, p. 1321; Setiamurti and Kurniawati, 2024; Yin et al., 2025).

However, multiple studies have shown that, in practice, creative writing courses are prone to information and stimulus overload (Barton et al., 2023, p. 1318; Forte-Celaya et al., 2021; Sibo et al., 2024). Cognitive load theory distinguishes cognitive load into intrinsic, extrinsic, and germane load. It also points out that when teaching involves unnecessary processing, it demands more working memory resources and weakens the learning effect (Sweller et al., 2011). In the process of creative writing conception, if the classroom presents high-density multimodal examples, it may unintentionally increase representational density and extrinsic load. The time that students have for internal simulation and free association may be compressed (Forsler et al., 2024; Graham et al., 2024; Le-Thi et al., 2020, p. 1219). In media and audio-visual-related majors, this structural problem is particularly prominent. They are more accustomed to “watching already-made stories” than to generating images from inner representation-mental imagery (Chen Q. et al., 2024; Chen X. et al., 2024; Hong et al., 2023; Jajdelska et al., 2019, p. 3). Mental imagery refers to perceptual-like experiences in the absence of external stimuli and supports the construction of internal scenes through visual and multisensory simulation (Kosslyn and Jolicœur, 2021). Mental imagery supports learning transfer and creative ideation; it is frequently discussed in theory as important for creativity and expression.

Therefore, the gap in this study is that current creative writing classes in higher education usually focus on increasing multimodal resources rather than strategically guiding internal imagery at the conception stage. Mental imagery is a key cognitive mechanism for creativity generation, but it is rarely emphasized in educational methods (Trypke et al., 2023, 2024).

Accordingly, to fill this gap, based on cognitive load theory and research on mental imagery, this study’s contribution and innovation are the proposal of the “leave space for imagination” framework, which uses single-modal prompts and the “generate first, then compare” sequence as an operational means to protect internal simulations during the early conception stage.

Based on this goal, this paper raises the following research questions.

*RQ1*: What design principles and task components define a single-modality, low-specification framework for supporting imagery generation in creative writing?

*RQ2*: Under the “Leaving Space for Imagination” structured intervention, do students’ VVIQ scores increase from pre-test to post-test over the 9-week instructional unit?

*RQ3*: Under the “Leaving Space for Imagination” structured intervention, do students’ PSIQ total and subscale scores increase from pre-test to post-test over the 9-week instructional unit?

Notably, RQ1 establishes the design foundation for the “Leaving Space for Imagination” intervention examined in RQ2 and RQ3, with the efficacy of this framework for enhancing VVIQ scores forming the core empirical focus of the study’s quantitative tests.

Theoretical contribution: Single-modality, low-specification tasks are defined as an imagery-support mechanism in creative thinking courses, offering an alternative to “more multimodal input is better” by protecting cognitive space for internal simulation.
Design contribution: Propose a scalable “four-stage lesson structure” that integrates the systems of image evocation, extension, preliminary expression, and sharing with peers into the regular classroom process.
Empirical contribution: The Vividness of Visual Imagery Questionnaire (VVIQ) and the Plymouth Sensory Imagery Questionnaire (PSIQ) were used as proximal psychological indicators to characterize within-subject pre–post changes associated with the implementation of the framework, motivating future controlled studies that incorporate comparison groups and performance-based outcomes.

## 2 Theoretical and design framework

### 2.1 Mental imagery and cognitive load in creative writing

Mental imagery is defined in cognitive psychology as the internal visual, auditory, and other sensory experiences without the influence of external stimuli (Gu et al., 2025; Kosslyn, 1990; Marks, 1973, 2023). In creative writing, mental imagery enables the writer to mentally simulate spatial relationships, sensory details, and emotional atmospheres (Gu et al., 2025; Miller, 2004), thereby supporting the construction of characters, scenes, and plot events. Then, these internal representations can be transformed into linear language (Huang et al., 2022, p. 32). Relevant studies have shown that the vividness of mental imagery is related to creativity and comprehension ability (Friedlander et al., 2022). The more vivid the mental imagery, the more it helps to construct a multi-level narrative world and sensory experience (Ye et al., 2024).

The cognitive load theory holds that working memory capacity is limited and that the complexity of the task, the presentation method, and prior knowledge jointly determine the learning outcome (Jiang et al., 2023, p. 389; Sweller, 1988, p. 274). Creative writing itself is a high-load thinking process, from conceiving story plots to arranging narrative segments, all of which bring high internal load; if such a high demand is presented in the classroom will significantly increase the additional load and distract students’ attention from operations (Sweller et al., 1998, p. 35).

In this context, resources for internal simulation and free imagination are compressed, making it easier for students to reproduce familiar patterns. From the perspective of the intersection between mental imagery research and cognitive load theory, creative writing does not necessarily benefit from continuously increasing external input; moderate “leaving space for imagination” may be more conducive to the operation of the mental imagery system (Friedlander et al., 2022, p. 2; Suvanto, 2016, p. 8; de Koning et al., 2016). When a task offers only sparse and open-ended clues rather than a complete picture, writers need to actively mobilize their experience and imagination to fill in the gaps (Healey, 2025; Kosslyn, 1990). This process itself is an effective investment in generative load (Healey, 2025; Zhu et al., 2023).

Building on this idea, single-modality, low-specification prompts can serve as instructional scaffolding that helps generate mental imagery in creative writing. By intentionally managing sensory channels and the amount of information provided, create the mental space learners need, making mental imagery generation not just a side effect but a necessary step in writing progress. The upcoming sections will explore specific types of single-modality tasks and how they are organized in classroom settings in line with this concept.

### 2.2 Single-modality prompts as imagery scaffolds

Transform single-modality, low-specification tasks into a classroom scaffold that can be precisely controlled. This aligns with the concept of “imagined reading” emphasized by previous researchers, which states that sparse clues prompt learners to construct scenarios and details beyond the text (Pelttari, 2017; Wright and Weible, 2024). It should be emphasized that a single modality does not equate to a lack of differentiation in support. On the contrary, the scaffold design needs to keep the clues and task difficulty within the learners’ tolerance range to avoid confusion or loss of engagement (Friedlander et al., 2022, p. 2; Karwowski et al., 2022, p. 250).

The single-modality scaffolds in this study primarily manifest in three forms: text-only prompts, image-only prompts, and audio-only prompts, which are triggered internally through different entry points.

Text-only prompts present brief scenarios or the beginnings of unfinished stories, without providing images or audio. Learners are required to construct internal scenarios, characters, and causal chains to advance the narrative (Chen Q. et al., 2024; Chen X. et al., 2024; Healey, 2025). To ensure operability, the prompts need to strike a balance between openness and comprehensibility; overly abstract cues may raise the threshold for conception rather than facilitating imagery generation.

Image-only prompts presenting a single or a group of static images, without subtitles or plot explanations, require students to infer the history and subsequent events and to fill in the gaps in space and time outside the shots (Aydın and Yildiz, 2024; Jajdelska et al., 2019, p. 7). Since highly type-specific or strongly culturally scripted, students may also fall into “template-based associations,” the selection of images should aim to maintain a certain degree of ambiguity, and avoid overly strong cultural script cues (Jajdelska et al., 2019, p. 7; Listyani, 2019).

Sound-only prompts only playing environmental sounds or soundscapes, without visual cues, students need to infer the scene and emotions “behind the sound” based on rhythm, tone, and spatial sense, and translate them into textual descriptions, providing a generation path from auditory perception for multi-sensory mental images (Quiroga-Martinez et al., 2024; Yue et al., 2023). To reduce the frustration of learners with weak auditory imagery, the task instructions provide brief guiding questions (such as “Where does the sound come from?,” “What emotion does this sound scene correspond to?”), rather than offering completely unguided open-ended explanations.

Regarding single-modality tasks as “mental image scaffolds” does not mean denying multimodal resources; rather, it advocates functional division at different learning stages. This sequence differs from the linear multimodal presentation, more closely aligning with the resource-allocation requirements of cognitive load theory (Fiorella et al., 2019; Fiorella and Mayer, 2021; Jiang et al., 2023, p. 388). Therefore, the core of its teaching approach is not merely to pursue a “single modality,” but rather to control the sequence and degree of representation, thereby preserving space for the growth of imagery vividness. The following sections will describe the design principles and framework.

### 2.3 Design principles and framework

To enhance the reusability and transferability of this approach, this framework clarifies it from three aspects: design principles, task families, and the four-stage classroom structure.

**Figure 1:**
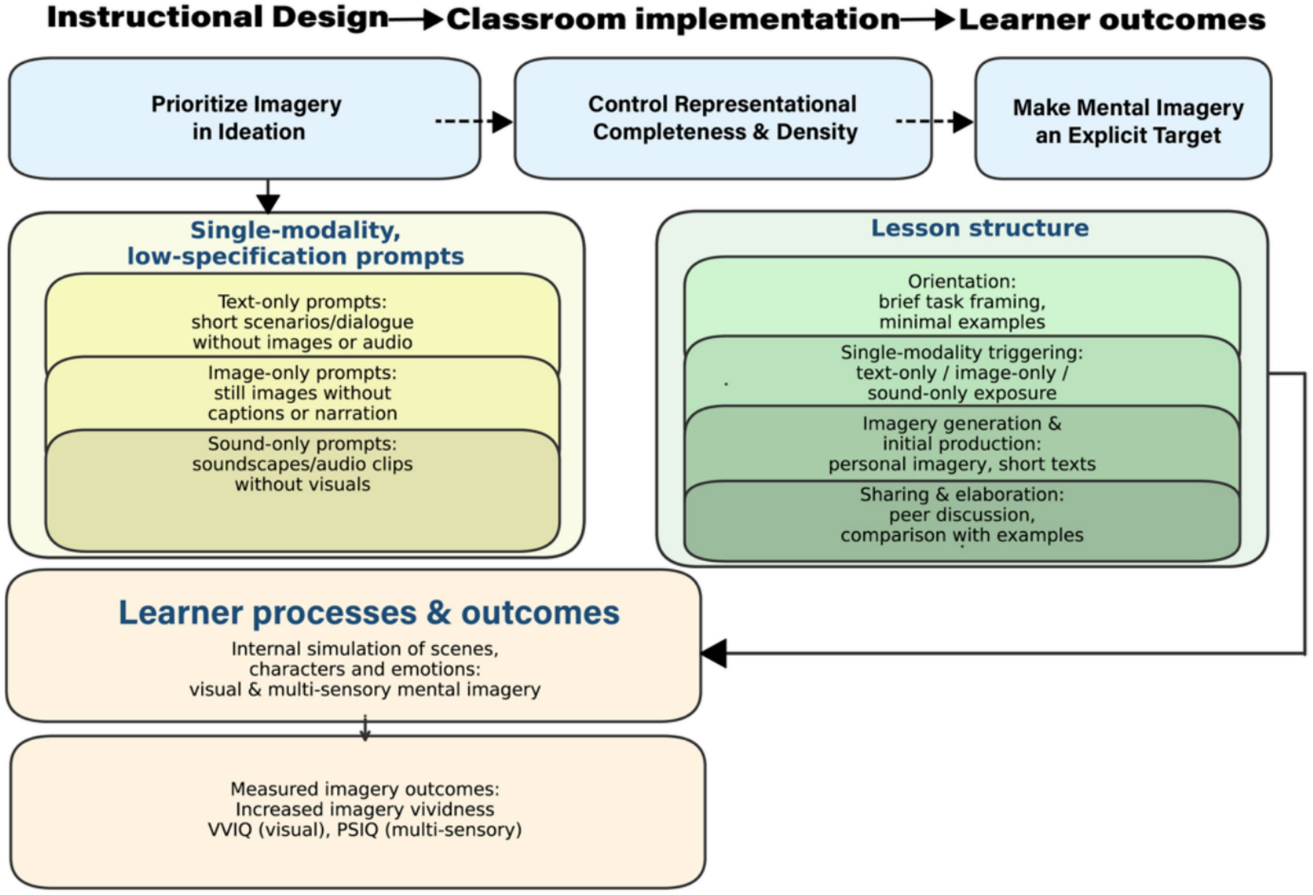
“Leaving Space for Imagination” imagery-informed teaching framework.

Figure 1 illustrates the imagery-oriented teaching framework, “Leaving space for Imagination,” and explains its operational logic.

Three interconnected design principles.

First, emphasize the creation of imagery during the ideation stage. Before story development and material creation, the presentation of examples and multimodal resources should be postponed, encouraging students to independently produce visual and multi-sensory imagery based on ambiguous clues (Fiorella and Mayer, 2021; Karwowski et al., 2022, p. 236).

Second, regulate the completeness and density of representation. According to cognitive load theory, task prompts should stay brief and open-ended, minimizing unnecessary explanations (Castro-Alonso et al., 2021; Leahy and Sweller, 2004, 2007; Zou et al., 2025).

Third, make mental imagery an explicit learning goal. Teachers should assist students in becoming aware of their own imagery experiences and in learning to manage and improve them through questioning, reflection, and peer discussion (Hall and Fishburne, 2010, p. 10; Paudyal, 2023, p. 124).

Guided by these principles, this framework emphasizes three types of single-modality, low-specification prompts, which are described in section 2.2. The main goal of these three task types is to preserve cognitive space for imagery creation through a single modality and low detail.

The framework shows the four-stage lesson structure. To ensure consistency in implementation, classroom procedures follow a stable sequence.

Stage 1—Task introduction: Provide a brief description of the writing goals and constraints, without including examples.

Stage 2—Single-modality trigger: Interact with text, image, or sound stimuli under the minimum necessary guidance.

Stage 3—Image generation and initial production: Transform the newly created images into short writings or situational descriptions, marking the first verbalization of imagery.

Stage 4—Sharing and elaboration: Through peer communication, compare different narrative paths; only introduce selected examples at this stage to support reflection and revision (Filosofi et al., 2024; Healey, 2025; Nicol and McCallum, 2021).

The expected learner process and outcomes of the framework. Internally, unimodal tasks trigger simulations of scenes, characters, and emotions, enhancing the vividness of visual and multisensory mental imagery (Friedlander et al., 2022, p. 5; Macfie et al., 2023, p. 173; Nánay, 2017, p. 130). This study measures this change through the VVIQ and PSIQ (Cui et al., 2007; Pérez-Fabello and Campos, 2020, p. 5). It should be emphasized that the present study did not measure the performance of writing works. Therefore, directions related to writing quality are regarded as future research directions.

### 2.4 Distinguishing the framework from existing mental imagery and creative writing models

Research on mental imagery generally posits that writers engage multisensory internal representations during the processes of conception and comprehension, and transform internal simulations into written expressions (Kosslyn, 1990; Nánay, 2017). Concurrently, teaching practices in creative writing often advance learning through examples, analyses of texts and fragments, and writing tasks (Jajdelska et al., 2019; Healey, 2025). However, there is a critical gap at the classroom design level: imagery is often regarded as a “natural concomitant process” of writing, yet it is rarely explicitly set as a trainable and operable teaching objective. Corresponding teaching often attempts to “spark inspiration” by continuously adding examples and multimodal materials, while neglecting that high-density representations may encroach upon the cognitive space required for internal simulations (Sweller, 1988; Leahy and Sweller, 2004, 2007; Mayer, 2024).

The “Leaving Space for Imagination” framework differs in two aspects. Firstly, the design stance shifts from “input superposition” to strategic withdrawal of representational support: in the early stages of conception, single - channel, low - prescriptive cues are used to reduce representational density, and the presentation of examples is postponed to minimize the risks of redundant processing and attention dispersion, thus prioritizing the occurrence of internal simulations (Sweller et al., 1998; Fiorella and Mayer, 2021; Karwowski et al., 2022). Secondly, the framework adopts a chronological configuration of “generate first, then compare”: learners first form self - generated representations, and examples are then introduced during the discussion/revision phase for comparison and refinement, enabling the alignment with norms to be based on existing internal representations (Fiorella et al., 2019; Fiorella and Mayer, 2021; Mayer, 2024).

## 3 Methodology

### 3.1 Research design

This study used a one-group pre-test–post-test quasi-experimental design to examine short-term, proximal changes in students’ self-reported mental imagery vividness as they integrated the “Leaving Space for Imagination” framework into an undergraduate creative writing course. Given the one-group design, the analyses focus on within-subject pre–post differences and do not support causal inference; future studies should incorporate comparison groups.

Participants were *N* = 100 undergraduate students from the art department of a regional university in China (male:female ≈ 7:3), majoring in digital media art, animation, and film directing. The participants were sophomore students aged 19 to 22. They were recruited through convenience sampling from the compulsory courses. All participants completed two rounds of assessments, and thus all were included in the statistics. The teaching unit was embedded in a required creative writing course, delivered to the whole class, and taught by the same instructor every 2 class hours per week and across 9 weeks.

After the teaching intervention, all participants who completed the pre-test and post-test were included in the analysis. Self-assessment questionnaires for VVIQ and PSIQ, as proximal self-report indicators, were administered before and after the intervention. Within-subject pre–post comparisons have been used in educational contexts to evaluate short-term changes in psychological indicators following instructional implementation.

RQ1 provides the foundational design basis for the “Leaving Space for Imagination” intervention empirically tested in the following hypotheses (H1–H3). They are correlated with RQ2 and RQ3, which serve as a direct quantitative test of the framework’s efficacy in enhancing mental imagery vividness.

*H1*: Under the “Leaving Space for Imagination” structured intervention, the post-test VVIQ score will be higher than the pre-test score.

*H2* (PSIQ total): Under the “Leaving Space for Imagination” structured intervention, PSIQ total scores will be higher at post-test than at pre-test.

*H3* (PSIQ subscales): Under the “Leaving Space for Imagination” structured intervention, each PSIQ subscale score will be higher at post-test than at pre-test.

Ethical approval was obtained at the institutional level, students signed informed consent forms, and their names were anonymized using codes, without any personal identification information. Regarding internal validity, observed pre–post differences may reflect factors such as testing effects, maturation, history, or regression to the mean, rather than the instructional framework alone; future research should incorporate comparison groups to strengthen causal inference and extend evaluation to additional outcomes where appropriate.

### 3.2 Instruments

#### 3.2.1 Vividness of Visual Imagery Questionnaire

The VVIQ is one of the questionnaires used in this study to assess participants’ visual imagery vividness. This questionnaire, developed by Marks (1973), consists of 16 items divided into four daily scenario dimensions. Participants are required to visualize an image during the test and then rate the clarity of their mental image on a 5-point scale (from “almost no image” to “close to a real visual experience”). The instrument has been widely used in imagery research, including studies linking individual differences in imagery vividness to cognitive outcomes (Friedlander et al., 2022; Marks, 1973).

In the present study, the VVIQ Item scores follow the original scoring procedure (1–5), yielding a total score of 16–80. A higher total score indicates more explicit visual imagery. This questionnaire serves as an indicator of the proximal effect of the “Leave Space for Imagination” teaching unit on visual channels in pre- and post-tests (Friedlander et al., 2022; Shaw and Belmore, 1982; Wright et al., 2024). The VVIQ was administered in Chinese. The instrument was translated into Chinese and back-translated into English by two independent bilingual researchers; discrepancies were resolved by consensus, and the wording was piloted with a small group of students to ensure clarity.

#### 3.2.2 Plymouth Sensory Imagery Questionnaire

The PSIQ questionnaire serves as a tool for measuring the vividness of multisensory imagery. This questionnaire was developed by Andrade et al. (2014). To assess multisensory imagery across 7 sensory dimensions, including vision, hearing, smell, taste, touch, bodily sensation, and emotional experience.

Participants are required to describe a mental scene for each item and rate each item on a scale from 0 (no imagery or sensation at all) to 10 (as vivid as a real-life experience), with a total score between 35 and 350. The higher the score, the more vivid the multisensory imagery. The total PSIQ score and its sub-dimension scores are used as indicators for evaluating the key proximal outcomes of multisensory teaching units (Andrade et al., 2014). The PSIQ was administered in Chinese. The instrument was translated into Chinese and back-translated into English by two independent bilingual researchers; discrepancies were resolved by consensus, and the wording was piloted with a small group of students to ensure clarity.

#### 3.2.3 Internal consistency in the present sample

It is important to assess their internal consistency in this sample. Based on pre-test item scores, Cronbach’s *α* was calculated for the 16 items of the VVIQ and the 35 items of the PSIQ. The results are presented in Table 1.

**Table 1:** Reliability coefficients for the VVIQ and PSIQ (pre-test, *N* = 100).

Instrument	Construct assessed	Cronbach’s *α*	*N* of items
VVIQ	Visual imagery vividness	0.922	16
PSIQ	Multi-sensory imagery vividness (total)	0.960	35

The *α* coefficient of VVIQ is 0.922, and that of the PSIQ total scale is 0.960. Both exceed the general standard of 0.70 and the “good reliability” level of 0.80 (Jankowska and Karwowski, 2023, p. 6). There was no significant improvement in “*α* after deleting this item” for each item, indicating that the items contributed equally.

### 3.3 Teaching procedure

This teaching unit follows the “four-stage lesson structure” shown in Figure 1, described in section 2.3 (Pérez-Fabello and Apostolou, 2024; Rosen et al., 2020). The teaching unit is carried out within the regular course schedule, lasting for 9 weeks. There are 18 classes, each lasting 45 min. The VVIQ and PSIQ were administered as pre-tests before the start of the teaching unit and as post-tests after its completion. Across the unit, three unimodal prompt types (text-only, image-only, and sound-only) were used in an alternating schedule, while the lesson structure remained constant. Every 2 lessons followed the same procedure.

1. Task briefing

Briefly explain the course objectives of the single-channel triggering method and the writing task for this class. Minimize the lecture, and the core requirement is “form the mental image first before writing” to reduce unnecessary external processing during the conception stage (Alwasilah, 2024, p. 3978; Healey, 2025).

1. Unimodal trigger

Present a single modality of information (text-only, image-only, and sound-only), provide a quiet environment, and sufficient time for students to construct an initial mental scene based on the clues. Maintain consistency in guidance under a low-specification framework, and use short directional questions (such as “Who/Where/What happened?” “What can be heard/felt?” “What emotion does this situation correspond to?”).

1. Imagery generation and initial externalization

Students complete short essays of varying lengths based on internally generated images. During this process, the instructor provides only time reminders and the minimum necessary process support (such as suggesting the addition of sensory details or causal chains), avoiding plot/structure templates or complete story frameworks (Drake et al., 2025; Healey, 2025; Miller, 2004).

1. Sharing and refinement

Students share snippets and describe the “sight/hear/feel” aspects during the conception process; peers and teachers’ feedback focuses on the clarity of imagery and sensory details, rather than forcing a convergence to a single “standard narrative.” Only a few examples are introduced at this stage for comparison, to support reflection and revision.

This process is repeatedly carried out in different themes to form a continuous practice of “image-driven conception - external expression.” To enhance consistency in implementation, the same sequence and guidelines are maintained across all classes. The above arrangement ensures that the pre-test and post-test align with the framework’s implementation cycle: the post-test is conducted immediately after the teaching unit is completed. The above operational description aims to support replication and transfer in similar writing courses (Paudyal, 2023, p. 123; Zhou et al., 2024). A concise session-by-session outline and the replicable task structure are provided in Appendix A.

### 3.4 Data analysis

All statistical analyses were conducted using SPSS 27. First, the pre- and post-test data were organized and checked for coding errors. Before performing inferential analysis, descriptive statistics were calculated for all scale totals and PSIQ subscales, and the distribution shapes were examined using histograms and key normality indicators to determine whether the paired-samples *t*-test was appropriate (Kvamme et al., 2024, p. 10).

At the same time, Cronbach’s *α* coefficients were computed for VVIQ and PSIQ using pre-test data to assess the scales’ internal consistency within this sample (Friedlander et al., 2022, p. 9; Kvamme et al., 2024, p. 10).

Primary inferential analyses were conducted to test H1–H3 by examining within-subject pre–post change patterns. Specifically, paired-samples *t*-tests compared pre-test versus post-test scores for VVIQ total (H1) and PSIQ total (H2) at *α* = 0.05 (two-tailed). Paired *t*-tests were also conducted for each of the seven PSIQ sensory subscales to further characterize multisensory change patterns consistent with H3; to control the familywise error rate across these subscale tests, *p*-values were adjusted using the Holm–Bonferroni procedure. For each paired comparison, the mean difference, 95% confidence interval of the difference, t statistic, degrees of freedom, and adjusted *p* value were reported.

To assess the practical importance of the findings, Cohen’s *dz* and its 95% confidence interval were calculated from the paired differences and interpreted according to standard thresholds for small, medium, and large effects (Maier et al., 2020, p. 5; Soland and Thum, 2019). Additionally, Pearson correlation coefficients were calculated between the pre- and post-test total scores of VVIQ and PSIQ to examine the relationship and stability of visual and multisensory imagery vividness, providing supplementary insights into the interpretation of the results.

## 4 Results

### 4.1 Data screening and sample inclusion

Prior to hypothesis testing, the dataset was screened for coding errors and out-of-range values. All VVIQ and PSIQ item responses fell within the permitted ranges, and all participants who completed both the pre-test and post-test were included in the paired analyses (*N* = 100).

### 4.2 H1: changes in visual imagery

*H1*: Under the “Leaving Space for Imagination” structured intervention, the post-test VVIQ score will be higher than the pre-test score.

This section highlights how the vividness of visual imagery changes over time. Table 2 displays the results of the paired-samples *t*-test for VVIQ scores.

**Table 2:** Paired-samples *t*-test for changes in VVIQ scores.

Measure	Pre-test M (SD)	Post-test M (SD)	Mean difference (post − pre)	95% CI for difference	*t*(99)	*p*	Cohen’s *dz*	95% CI for *dz*
VVIQ	46.54 (10.58)	58.02 (12.05)	11.48	(10.25, 12.72)	18.45	<0.001	1.85	(1.52, 2.17)

M, mean; SD, standard deviation; CI, confidence interval; *t*, *t* statistic; *p*, probability value.

As shown in Table 2, the mean post-test VVIQ score (M = 58.02, SD = 12.05) was higher than the mean pre-test score (M = 46.54, SD = 10.58). In this study, the difference was defined as “post-test − pre-test,” and the average difference of 11.48 [95% CI (10.25, 12.72)] indicates an overall improvement of about 11.5 points in students’ visual imagery vividness. The results of the paired-samples *t*-test showed that this improvement was highly statistically significant, *t*(99) = 18.45, *p* < 0.001, with a 95% confidence interval (10.25, 12.72). Consistent with H1, the results indicate a robust within-subject pre–post increase in self-reported visual imagery vividness over the 9-week teaching unit. To quantify practical significance, the paired-design effect size, Cohen’s *dz*, was computed from the paired differences. The effect size was large [*dz* = 1.85, 95% CI (1.52, 2.17)].

### 4.3 Changes in multi-sensory imagery

#### 4.3.1 H2: PSIQ total pre–post

*H2* (PSIQ total): Under the “Leaving Space for Imagination” structured intervention, PSIQ total scores will be higher at post-test than at pre-test.

We used a paired-samples *t*-test to compare total PSIQ scores before and after the test (*N* = 100) to test hypothesis H2. As shown in Table 3, the total PSIQ score after the test (mean = 202.48, standard deviation = 25.94) was higher than that before the test (mean = 182.14, standard deviation = 23.40), with an average increase of 20.34 points [95% confidence interval (17.64, 23.04)]. The paired-sample *t*-test indicated that this pre-test-post-test difference was statistically significant, *t*(99) = 14.93, *p* < 0.001. Consistent with H2, the results showed that at the total score level, there was a significant pre-test-post-test increase in self-reported multi-sensory imagery vividness. The effect size in the paired design was large [Cohen’s *dz* = 1.49, 95% confidence interval (1.21, 1.78)].

**Table 3:** Paired-samples *t*-test and effect size for PSIQ total score (*N* = 100).

Measure	Pre-test M (SD)	Post-test M (SD)	Mean difference (post − pre)	95% CI for difference	*t*(99)	p	Cohen’s *dz*	95% CI for *dz*
PSIQ total	182.14 (23.40)	202.48 (25.94)	20.34	[17.64, 23.04]	14.93	<0.001	1.49	[1.21, 1.78]

M, mean; SD, standard deviation; CI, confidence interval; *t*, *t* statistic; *p*, probability value.

#### 4.3.2 H3: PSIQ subscales pre–post

*H3* (PSIQ subscales): Under the “Leaving Space for Imagination” structured intervention, each PSIQ subscale score will be higher at post-test than at pre-test.

To test H3, a paired-sample *t*-test was conducted for each of the seven PSIQ sensory subscales (visual, auditory, olfactory, gustatory, tactile, bodily sensation, and emotional imagery). Table 4 summarizes the descriptive statistics of the pre-test and post-test, while Table 5 reports the adjusted paired *t*-test results after Holm–Bonferroni correction to control the overall error rate in the comparison of the seven subscales (Woelk et al., 2022).

**Table 4:** Descriptive statistics for PSIQ subscale at pre-test and post-test.

PSIQ measure	*N*	Pre-test M (SD)	Post-test M (SD)
Visual imagery	100	25.40 (3.53)	28.92 (3.99)
Auditory imagery	100	25.06 (3.56)	28.74 (4.05)
Olfactory imagery	100	25.17 (3.59)	28.80 (4.06)
Gustatory imagery	100	25.47 (3.67)	29.20 (4.01)
Tactile imagery	100	25.52 (3.69)	29.15 (4.36)
Bodily sensations imagery	100	25.22 (3.84)	28.93 (4.23)
Emotional imagery	100	25.24 (3.62)	28.74 (4.13)

M, mean; SD, standard deviation. PSIQ subscale scores are reported as raw sum scores ranging from 0 to 50 (sum of five items), and the PSIQ total score ranges from 0 to 350.

**Table 5:** Paired-samples *t*-tests and paired effect sizes (*dz*) for PSIQ subscale changes (*N* = 100).

PSIQ subscale	Mean difference (post − pre)	*t*(99)	p	*p* (Holm–Bonferroni)	Cohen’s *dz*
Visual imagery	3.52	16.76	<0.001	<0.001	1.68
Auditory imagery	3.68	16.31	<0.001	<0.001	1.63
Olfactory imagery	3.63	16.26	<0.001	<0.001	1.63
Gustatory imagery	3.73	16.22	<0.001	<0.001	1.62
Tactile imagery	3.63	16.26	<0.001	<0.001	1.63
Bodily sensations imagery	3.71	16.18	<0.001	<0.001	1.62
Emotional imagery	3.50	15.59	<0.001	<0.001	1.56

Holm–Bonferroni adjustment was applied across the seven subscale tests to control the familywise error rate.

**Figure 2:**
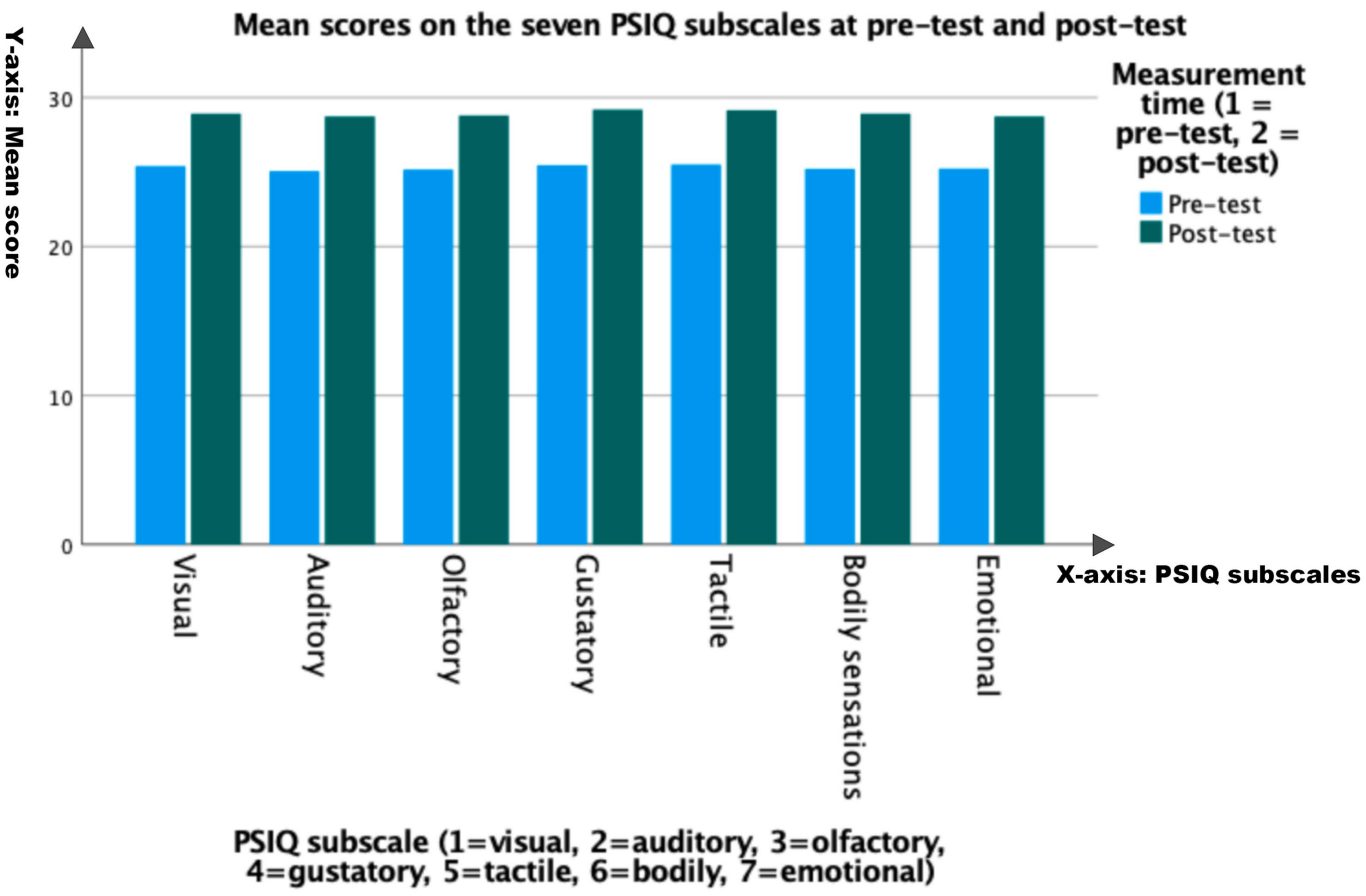
Mean scores on the seven PSIQ subscales at pre-test and post-test.

As shown in Table 5 and Figure 2, all seven sub-scales showed significant changes before and after, and these results remained significant after Holm–Bonferroni correction (all adjusted *p*-values were <0.001), supporting Hypothesis 3. The effect sizes for each sub-scale were large (*dz* = 1.56–1.68), indicating significant standardized differences in the clarity of multisensory imagination across sensory channels before and after. These results describe the consistency of the changes before and after the implementation of the teaching unit, but, given the single-group pre-test–post-test design, they cannot determine causality.

## 5 Discussion

### 5.1 Pedagogical value and interpretive boundaries of the “Leaving Space for Imagination” framework

The “Leaving Space for Imagination” framework described in this study combines single-modality, low-specification value cues with explicit mental imagery goals into a practical four-stage classroom process. Across the short teaching unit, students’ self-reported visual and multisensory imagery vividness showed clear pre–post increases with large paired-design effect sizes. This is consistent with reducing external input, managing information density, and Leaving space for idea development may preserve cognitive space for internal simulation and imagery generation (Fiorella et al., 2019; Fiorella and Mayer, 2021; Sibo et al., 2024).

However, caution should be exercised when interpreting the effects of this framework. Since the study used a single-group pre-test–post-test design, improvements in scores could be influenced by the passage of time or test familiarity (McVeigh et al., 2023; Ramalingam et al., 2020). Therefore, a more accurate conclusion is that this framework is linked to improved mental imagery vividness, rather than establishing that each element has a direct causal effect. Moreover, this study focused on a proximal indicator of imagery vividness and did not simultaneously examine systematic changes in the quality of the work, so whether enhanced mental imagery can lead to better text quality remains to be investigated.

### 5.2 Visual imagery: implications of reduced representational density during ideation

The VVIQ results showed a robust pre–post increase in self-reported visual imagery vividness over the teaching unit. The observed pre–post increase in VVIQ scores is consistent with the possibility that placing “imagery first” at the ideation stage and reducing representational density may encourage learners to engage more actively in generating and inspecting internal visual representations. However, alternative explanations (e.g., test familiarity, maturation, concurrent course experiences) cannot be ruled out in the present design (Antonietti and Colombo, 2011; Friedlander et al., 2022, p. 1; Polland, 1996, p. 30; Suggate and Lenhard, 2022).

However, “leaving space” are not equally effective for all learners: students with a stronger foundation in visual mental images demonstrate greater improvement, while those with a lower initial ability may need extra prompts or gentle structured support (Cavazos et al., 2025; Le-Thi et al., 2020, p. 1239; Spagna et al., 2023, p. 150). Therefore, this framework is better suited for a step-by-step approach, allowing students with different levels of mental imagery to engage in learning without overloading their cognitive resources.

### 5.3 Multisensory imagery: breadth of pre and post–change and boundaries of inference

In this study, PSIQ total and all seven subscales increased from pre-test to post-test over the unit, indicating broad within-subject change patterns in self-reported multisensory imagery vividness. This indicates that the framework’s unimodal prompts were associated with broad changes in self-reported multisensory imagery vividness rather than changes restricted to a single channel (Nánay, 2017, p. 130). These cues are presented in a single mode when input, but require learners to construct a complete scene (location-activity-emotion) during the internal simulation. Scene construction typically involves multiple sensory and emotional components (such as olfactory, gustatory, tactile, bodily sensations, and emotional tone) beyond the presented mode. These components may be reflected in the PSIQ sub-scales that are not directly prompted. Importantly, since all results are self-reported, these patterns may also reflect general changes in imagination awareness or reporting style rather than specific pattern transfer.

One plausible interpretation is that when learners construct scenarios from sparse cues, they may recruit multiple sensory and affective components (e.g., sound, bodily sensations, emotional tone) as part of internal simulation, consistent with multisensory imagery accounts of scene construction (Nánay, 2017).

However, this study only reports the proximal outcome, psychological intention, and has not demonstrated whether it directly improves work quality or long-term creative performance. Therefore, the most defensible interpretation is that the framework was associated with short-term increases in self-reported vividness of multisensory imagery, a proximal psychological indicator. Whether such changes transfer to writing processes, motivation, or product quality remains an open question for future work.

### 5.4 Limitations and future directions

This study also has several limitations. First, the study adopted a single-group pre- and post-test design, and the sample was from the same institution, which limited both the internal validity and external generalizability to some extent (Cheung et al., 2025; Valosek et al., 2021).

Second, the core data mainly came from self-report questionnaires such as the VVIQ and PSIQ, and the direct relationship between improvements in mental imagery and the quality of creative works has not been systematically tested (Friedlander et al., 2022; Jankowska and Karwowski, 2015; LeBoutillier and Marks, 2003).

Third, the study did not include indicators of the ideation process, and there is a lack of fine-grained evidence on students’ strategy use, time allocation, and revision trajectories during the tasks (Guarnera et al., 2019; van de Kamp et al., 2016; May et al., 2020).

Based on this, future research can further explore the more complete mechanism chain relationship between psychological indicators, process behaviors, and ultimately creative products. This can be achieved through methods such as combining standardized writing tasks, using validated scoring standards for expert evaluation, and conducting longitudinal tracking. Additionally, as the application of generative artificial intelligence in writing teaching becomes increasingly common, it may be valuable to study how to integrate artificial intelligence-assisted feedback without replacing the imagination generated by learners themselves.

For universities, especially those with media and audio - visual related majors, the evidence provided by this study shows that it is possible to alleviate to some extent the structural contradiction of “excessive visual input and poor internal mental imagery,” providing a specific and feasible starting point for more targeted creative writing and film and television scriptwriting teaching reforms in the future.

## 6 Conclusion

This study proposes the “Leaving Space for Imagination” framework. Using single-modality low-specification prompts during the ideation stage reduces the representation density and reserves the necessary cognitive space for learners to simulate their internal situations. The basic idea is consistent with the principles of cognitive load and mental imagery (Sweller et al., 2011; Mayer, 2024). This framework organizes teaching through a reproducible classroom micro-cycle of “Task briefing → Unimodal trigger (text-only, image-only, and sound-only) → Imagery generation and initial externalization → Sharing and refinement,” and explicitly sets the vividness of imagery as a proximal learning objective. Under the single-group pre-test–post-test design, the study observed that students showed improvement in both VVIQ and PSIQ, and the various dimensions of PSIQ maintained a consistent trend after Holm–Bonferroni correction. It should be emphasized that these results provide only preliminary evidence of a concurrent association with the teaching unit and do not support causal inference; alternative explanations, such as test familiarity, maturity effects, and concurrent learning experiences, may still exist. Future research should introduce a control group and delayed measurement, and increase the number of writing behavior/performance indicators to examine whether changes in vividness of imagery transfer to writing performance and further clarify the underlying mechanism.

## Data Availability

The raw data supporting the conclusions of this article will be made available by the authors, without undue reservation.

## Appendix A: Instructional unit overview and replication parameters

The intervention was delivered as a 9-week unit comprising nine 90-min sessions. Each session followed a fixed four-step routine designed to preserve cognitive space for internal simulation: Visual cue → Auditory cue → Dramatic-situation card → Integrative writing.

### Session routine (replicable template)

1. Visual cue (single-modality, low specification): brief exposure to a minimal visual stimulus to elicit internal visual representation; students record 2–3 visual anchors.
2. Auditory cue (single-modality, low specification): short sound cue/soundscape; students record 2–3 auditory anchors.
3. Dramatic-situation card (minimal constraint): a concise narrative constraint aligned with the session theme; students state a one-sentence scene goal and one observable cause–effect link.
4. Integrative writing (imagery-to-text externalization): time-boxed drafting of a micro-scene/scene fragment (typically 120–180 words), followed by an imagery-focused revision based on anchor clarity.

### Session sequence (themes)

Week 1 introduced the routine and administered pre-tests; weeks 2–8 implemented plot-type themes (overcoming the monster; the quest; rags to riches; tragedy; comedy; rebirth; voyage and return); week 9 emphasized consolidation (peer review and reduced prompting) and administered post-tests.

### Fidelity indicators

Implementation fidelity was supported by verifying that each session: (a) adhered to the four-step routine, (b) maintained low-specification cueing in steps 1–2 (avoiding early exemplar stacking), and (c) required imagery anchors to be recorded prior to drafting.
